# Flooding tolerance of four floodplain meadow species depends on age

**DOI:** 10.1371/journal.pone.0176869

**Published:** 2017-05-03

**Authors:** Johannes P. Gattringer, Tobias W. Donath, R. Lutz Eckstein, Kristin Ludewig, Annette Otte, Sarah Harvolk-Schöning

**Affiliations:** 1 Division of Landscape Ecology and Landscape Planning, Research Centre for Biosystems, Land Use and Nutrition (IFZ), Justus-Liebig-University Giessen, Giessen, Germany; 2 Department of Landscape Ecology, Institute for Natural Resource Conservation, Kiel University, Kiel, Germany; 3 Department of Environmental and Life Sciences, Biology, Karlstad University, Karlstad, Sweden; Helmholtz Centre for Environmental Research — UFZ, GERMANY

## Abstract

Numerous restoration campaigns focused on re-establishing species-rich floodplain meadows of Central Europe, whose species composition is essentially controlled by regular flooding. Climate change predictions expect strong alterations on the discharge regime of Europe’s large rivers with little-known consequences on floodplain meadow plants.

In this study, we aim to determine the effects of flooding on seedlings of different ages of four typical flood meadow species. To this end, we flooded seedlings of two familial pairs of flood meadow species of wetter and dryer microhabitats for 2 weeks each, starting 2, 4, 6, and 8 weeks after seedling germination, respectively.

We show that a 2-week-flooding treatment had a negative effect on performance of seedlings younger than 6 weeks. Summer floods with high floodwater temperatures may have especially detrimental effects on seedlings, which is corroborated by previous findings. As expected, the plants from wet floodplain meadow microhabitats coped better with the flooding treatment than those from dryer microhabitats.

In conclusion, our results suggest that restoration measures may perform more successfully if seedlings of restored species are older than the critical age of about 6 weeks before a spring flooding begins. Seasonal flow patterns may influence vegetation dynamics of floodplain meadows and should, therefore, be taken into account when timing future restoration campaigns.

## Introduction

Natural floodplains are among the ecosystems with the highest biodiversity on earth [1,2]. Their azonal vegetation is shaped by a broad hydrological gradient, regular flooding and soils of diverse composition, resulting in high habitat and species diversity [3]. Floodplain vegetation is also strongly influenced by humans [4,5]. Species-rich floodplain grassland, in particular, plays a crucial role in maintaining regional biodiversity but has also experienced a dramatic decline in Central Europe [6] mainly due to altered hydrological conditions through river training [7]. In particular floodplain meadows are amongst the most threatened plant communities in Europe [6,8]. They harbor typical and often endangered flood meadow species, also called river corridor plants, which are adapted to the specific disturbance regimes of floodplains [9]. To maintain the diversity of these species rich *Cnidion dubii* grasslands, protected by the EU Habitats Directive (Council Directive 92/43/EEC, habitat type 6440: alluvial meadows of river valleys of the *Cnidion dubii*) numerous restoration measures, mainly focusing on the reestablishment of rare species, have been conducted along the Rhine and Elbe Rivers, e.g. [10,11].

A challenge for such restoration projects is to consider and incorporate the effects of regular flooding, which represents a key factor in these dynamic floodplain meadows [12,13]. Plant species zonation of these grasslands is mainly driven by hydrologic conditions and land use [14–16] but also on a micro-habitat scale flood sensitive species are located on elevated microsites, whereas species with higher flooding resistance occupy depressions [17,18]. Flooding promotes recruitment of less competitive species through creation of open soil patches and suppression of flood-sensitive competitors [19] and plays a crucial role for maintaining diversity of rare species through recruitment of seedlings from the soil seed bank [20,21].

Additionally, vegetation dynamics are strongly driven by inter-annual-variation of flooding and drought [22]. Van Eck et al. [23] showed that summer flooding predominantly determines plant zonation in flood meadows, due to the more intense impact of summer floods vs. winter floods on plants. Hence, the timing of flooding events in relation to the plant life cycle strongly influences the occurrence and distribution of plant species. In particular, seedling establishment is the critical phase in the life cycle of many plants due to high mortality through diseases, injuries, and flooding or water deficit [24]. The age of seedlings at which these are exposed to flooding may play a crucial role for survival [25] and higher flooding tolerance may be related to species specific growth timing [26]. Nabben et al. [27] studied the effect of flooding on juvenile vs. mature *Rumex* plants (i.e. 5 vs. 14 weeks after germination) and confirmed the higher flooding tolerance of two-months-older plants. Since the timing of flooding events during the life cycle is of crucial importance for survival, a shift in the flooding regime might have large consequences. Other experimental studies focused on the duration of flooding events but did not take the age of plants into consideration [13,17,23,28].

Flooding dynamics in present-day floodplains are highly transformed by humans and through ongoing climate change. Multiple anthropogenic stressors and their impacts on flow regime are hard to distinguish and quantify [5,16]. Direct human alterations of rivers, such as construction of dams and dikes, trigger changes in water level fluctuations, which lead to alterations in terrestrial plant species composition [29]. Furthermore, possible large-scale floodplain restoration, such as dike relocation projects or ecological flooding (also known as managed flooding) [30,31] could additionally alter hydrological conditions of floodplain meadows [32] and subsequently their terrestrial plant diversity.

In addition, effects of climate change are supposed to alter plant species diversity in Central Europe [33] through altered discharge regimes of rivers [34]. For the river Rhine, a seasonal change of the discharge regime with increasing discharge in winter and decreasing discharge in summer is projected for the current century [35–37]. Additionally, intensity and frequency of extreme discharge events will increase [36,38]. Accordingly, zonation of floodplain ecosystems and similarly plant composition of flood meadow habitats might change through these multiple alterations in the discharge regime [39].

These alterations might also influence sediment deposition in the floodplain, since their soil composition strongly depends on frequency and magnitude of flooding events [40]. Models project considerable changes in sedimentation regime for the Rhine basin already within the current century [41]. Generally, sandy sediments can be found closest to the river channel whereas sites further away from the river are characterized by sediments with higher amounts of clay and organic matter [40,42]. Changes in these patterns may also influence vegetation since, e.g., the growth of woody floodplain plants depends on soil composition and is limited on coarse substrates after water table alterations [43]. However, effects of sediment grain size have not been studied with respect to flood meadow species in an experimental setup so far.

In summary, the increased unpredictability of habitat conditions under climate change induced shifts in the flow and sediment regime may act as obstacles for floodplain meadow restoration. To tackle this problem, the effects of flooding on survival and performance of plants should be investigated in more detail, to ensure success of future restoration campaigns. A recent study on flooding tolerance of wetland plants suggests that seasonal timing of flooding events plays a crucial role in flooding tolerance [44]. While the effects of flooding on adult plants have been studied before [13,17,18,23], studies of seedling establishment are scarce (but see Nabben et al. [27]).

Therefore, we investigate the impact of a 2-week flooding period on seedlings of different age (i.e. between 2 and 8 weeks after germination) of four characteristic species of flood meadows. To this end, we employed a completely randomized multi-factorial experiment to elucidate the impact of the factors species, microhabitat, seedling age, and soil composition on the performance of seedlings. We inundated seedlings of different ages and analyzed the impact of different factors on seedling survival and establishment.

Specifically, we tested the following hypotheses: Under a 2-week flooding period,

1. older seedlings perform better than younger seedlings,
2. the performance of seedlings decreases with increased sand content, and
3. plant species from wet microhabitats perform better compared to those of dry microhabitats.

## Materials & methods

### Study species

We chose two familial pairs of floodplain meadow species with preference for wetter and dryer microhabitats: *Sanguisorba officinalis* L. and *Veronica maritima* L. vs. *Sanguisorba minor* Scop. and *Veronica teucrium* L. (Table 1, The plant species nomenclature follows Jäger [45]). This balanced design avoids phylogenetic bias of the results [46]. All four species are perennials typically occurring on floodplain meadows along the Upper Rhine valley. The species characteristic of dryer microhabitats typically grow on slightly higher elevation than the species of wetter microhabitats. The plant species’ preferences for wetter and dryer micro niches are underlined by their Ellenberg indicator values (EIV) for moisture (F value, EIV m in Table 1) [47]. The species are target species in floodplain meadow restoration projects along the northern Upper Rhine [10]. In this experiment, they serve as umbrella species in the sense of Groom et al. [48] for the plant community of the *Cnidion dubii* meadows [49]. Here, that also comprises species from the EU Habitats Directive Annex I habitat type 6510: Lowland hay meadows. Seed material of a producer of autochthonous seeds (Rieger-Hofmann GmbH, Blaufelden-Raboldshausen, Germany) was used for the experiment.

**Table 1:** Differences in the survival of four floodplain meadow species among five age groups.

species	family	micro-habitat	EIV m	chisq	df	p	survival differences				
							age2	age4	age6	age8	noFl
*Sanguisorba officinalis* L.	Rosaceae	wet	7 ~	31.5	4	**<0.001**	a	a	b	c	b
*Sanguisorba minor* Scop.	Rosaceae	dry	3	94.5	4	**<0.001**	a	b	c	d	c
*Veronica maritima* L.	Plantaginaceae	wet	8 ~	0.0	4	1	a	a	a	a	a
*Veronica teucrium* L.	Plantaginaceae	dry	3	66.2	4	**<0.001**	ab	a	c	b	c

Differences were tested using a Wilcoxon-Mann-Whitney test (chi-square statistic), and subsequently, each paired combination was tested using a log-rank test with scores of Sun [50] for interval censored data (Z statistic). Four groups differed in seedling age at start of flooding period (age2-age8) and one group was the unflooded control (noFl). EIV m, Ellenberg indicator value for moisture;

^~^, indicator for alternating moisture conditions (F value, Ellenberg et al. [47]); chisq, chi-square value; df, degrees of freedom; p, error probability; p values < 0.05 are in bold; survival differences, significant differences (p < 0.05) in survival of plants between age groups according to log-rank test; for each species-seedling age combination: n = 20.

### Experimental design

The experiment was carried out from March to July 2015. The combination of four species, two types of soil composition, and five age groups (four groups differing in seedling age at start of flooding period, and one unflooded control) with ten replicates per combination resulted in a total number of 400 experimental plants. Seeds were cold-wet stratified for 21 days at 3°C in trays with potting soil in a climate chamber (Rumed type 3401; Rubarth Apparate GmbH, Laatzen, Germany).

Seeds germinated after 7 days (*V*. *teucrium* and *S*. *minor*) and after 10 days (*V*. *maritima* and *S*. *officinalis*) in a greenhouse (20°C by day / 15°C by night; photoperiod: 12 hours daily). Eleven days after germination 100 plants of every species, having almost the same size, were planted into pots (diameter: 9 cm on top, height: 7.8 cm). All these 400 plants had the same age of 11 days due to synchronous germination on day one.

Half of the plants were planted in a mixture of standard potting soil (F.-E. Typ P, HAWITA Gruppe GmbH, Vechta, Germany) and sand with a ratio of 3:1 and the other half in a soil:sand mixture of 1:1. We obtained nutrient equivalency in both soil treatment levels by adding slow release osmocote (Osmocote Exact Standard 3-4M, Everris International B.V., Geldermalsen, The Netherlands; 7.1% NO_3_-N, 8.9% NH_4_-N, 9% P_2_O_5_, 12% K_2_O) to the pots. With respect to Hidding et al. [44] we choose an intermediate nutrient scenario for this experiment with an osmocote equivalence (i.e. nutrients in standard potting soil + osmocote) of 100 grams osmocote per square meter.

At day 15 after germination, each of the 400 pots were placed inside a 1.2 L transparent polypropylene cup (diameter: 11.4 cm on top, height: 17 cm) and randomly distributed on a paved area at the research station Linden-Leihgestern (Hesse, Germany, UTM: 32U 478260 5598300, S1 Fig). Plants were placed under a rain shelter (height: 0.6 m, PE greenhouse grid film “Original Delta Folie SUV”) to avoid accidental flooding of the cups by precipitation. Under regular growth conditions plants were watered according to their daily demand (approx. 20–50 mL day^-1^).

**Fig 1:**
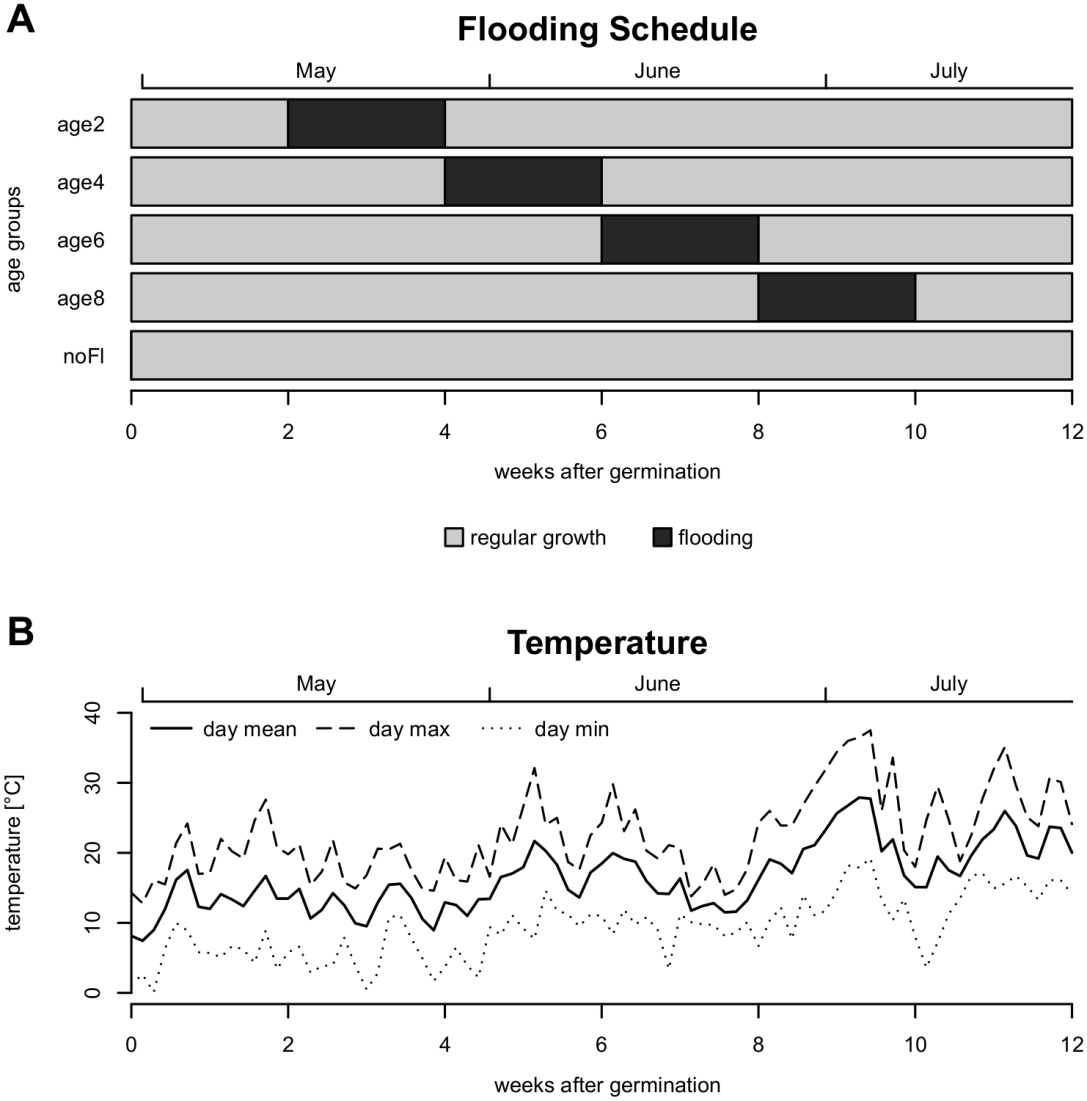
Time schedule and air temperatures for the flooding experiment of four floodplain meadow species. (A) Time schedule of age groups: four age groups with a 2-week flooding treatment starting 2, 4, 6, and 8 weeks after germination (age2, age4, age6, age8) and one unflooded control group (noFl) with regular growth through 12 weeks. (B) Temperature trend during time of the experiment (solid line: daily mean temperature, dashed line: daily minimum temperature, dotted line: daily maximum temperature). Temperature data from HLNUG (Hessian Agency for Nature Conservation, Environment and Geology, http://www.hlnug.de), weather station Linden (distance from experimental site: 700 m).

To test the response of seedlings of different age to a 2-week flooding period we performed five different treatments. Four groups of seedlings were flooded 2, 4, 6, and 8 weeks, respectively, after germination (age2, age4, age6, age8). One control group (noFl) was grown for 12 weeks without any flooding (Fig 1A). The flooding procedure comprehended 2 weeks of complete inundation: the cups each with one plant pot inside were filled completely with tap water (S1 Fig). Water levels were kept constant during the flooding period.

Survival (dead or alive) was assessed every 2 to 3 days based on physical appearance of plants: plants with green, turgid leaves and green buds were regarded as alive [27]. Total height of the plants and number of leaves were measured at the end of the experiment, i.e. after 12 weeks. We quantified specific leaf area (SLA) and aboveground biomass from measurable and living plants. For SLA, three fully expanded leaves with average size were collected of every plant, scanned and leaf area was measured with the software ImageJ [51]. The leaves were dried (48 hours at 60°C) and weighed, SLA was calculated as leaf area per leaf dry mass (m^2^·kg^-1^). Aboveground biomass was dried (24 hours at 100°C) and weighed and the biomass of the three leaves (SLA measurement) was added. Temperature data was obtained from Hessian Agency for Nature Conservation, Environment and Geology, weather station Linden (distance from experimental site: 700 m) [52].

### Analysis

In a first analysis, we tested the effects of seedling age on the cumulative seedling survival of the four species separately. To this end, a Kaplan-Meier survival analysis for interval censored data was done (i.e. measurements were taken at intervals of 2 to 3 days) [53]. We computed the non-parametric maximum likelihood estimate for the distribution from interval censored data to plot cumulative survival distributions for each species-seedling age combination with the R-package *interval* [54]. To test for differences among species, we calculated a Wilcoxon-Mann-Whitney test with generalized Wilcoxon-Mann-Whitney scores (chi-square statistic). Subsequently, differences between treatments were tested applying a log-rank test, which uses the most commonly used log-rank scores for right-censored data and reduces to the scores of Sun [50] for interval censored data (Z statistic).

In order to evaluate the effects of species, microhabitat, seedling age, and soil composition on survival of the plants, we computed accelerated failure time models [55]. We compared whether results from these analyses, containing all 400 plants, showed similar results as ANOVAs with only survived plants (n = 259). We fitted models with six error distributions (i.e. Weibull, exponential, gaussian, logistic, log-normal and log-logistic) of which the Weibull distribution, able to deal with non-constant hazards, produced the minimum error deviance and thus was preferred (function *survreg*, R-package *survival* [56]). The scale parameter of this analysis describes the form of the hazard function: scale parameter < 1: risk of death decreases with time; scale parameter > 1: risk of death increases with time [57]. To rule out other effects on survival (i.e. plant height and number of leaves before beginning of treatment) we computed Wilcoxon-Mann-Whitney tests, which did not show differences in plant height or number of leaves between surviving and dead plants.

In a next analysis, we tested for importance of the above factors on response variables: plant height, number of leaves, biomass and SLA of survived individuals using ANOVAs. We excluded dead plant individuals from this analysis to avoid detrimental effects of zero values on ANOVAs. Before analysis, the variables plant height, number of leaves and biomass were standardized using a natural logarithmic response ratio (*RR*) as suggested by [58].

$$RR=\text{ln}\left(P_{T}/\bar{P_{C}}\right)$$ (1)

**Fig 2:**
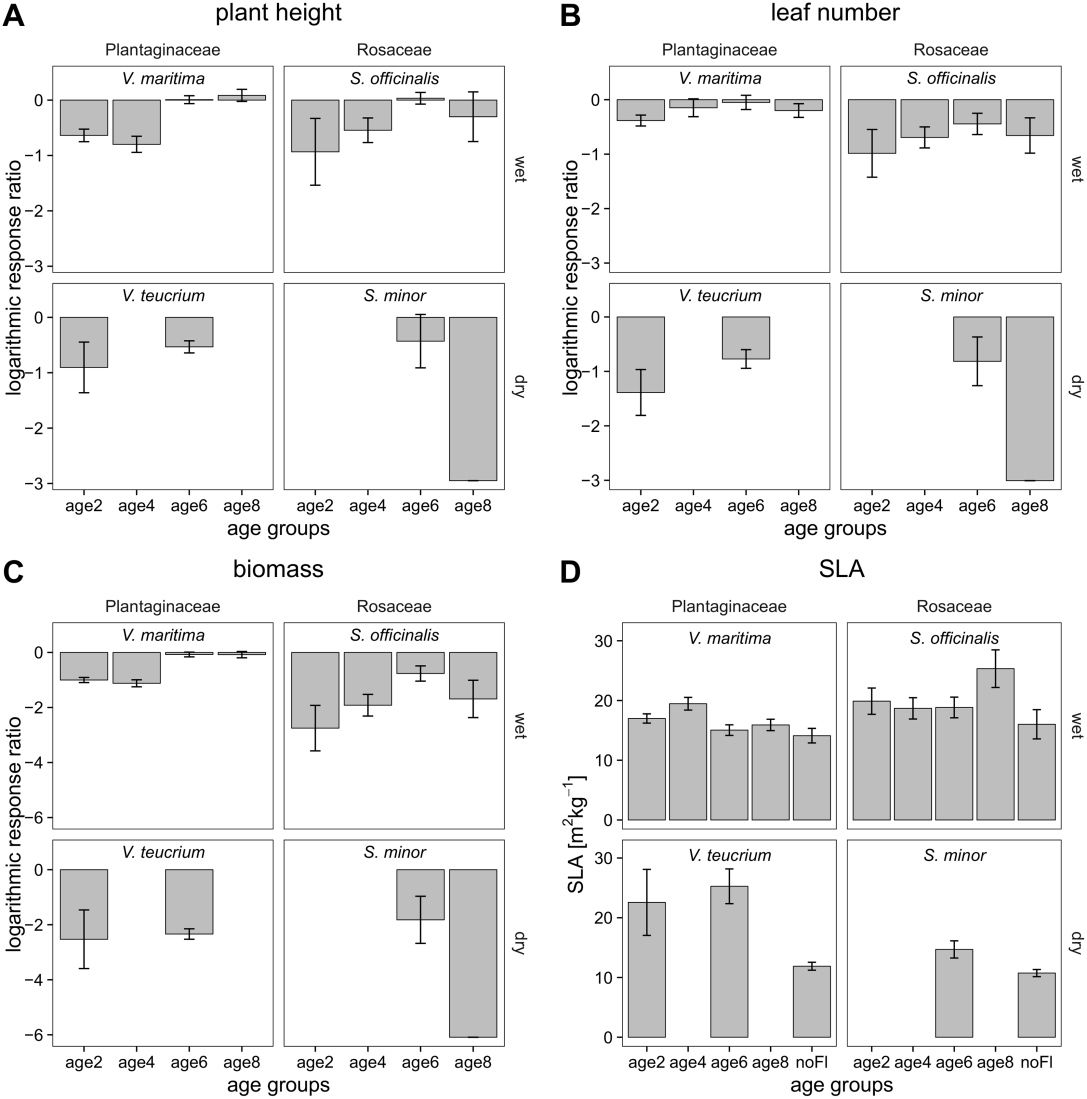
Performance of four floodplain meadow plant species after a 2-week flooding period. Mean (± 95% confidence interval) logarithmic response ratio of plant height (A), leaf number (B) and biomass (C), and mean (± 95% confidence interval) specific leaf area (SLA, D) for each species-seedling age group combination: *Veronica maritima* L., *Veronica teucrium* L., *Sanguisorba officinalis* L., and *Sanguisorba minor* Scop.; flooding started 2, 4, 6, and 8 weeks after germination (age2-age8), and control group with no flooding (noFl). Effects of flooding treatments on survived plants were considered significant (i.e. different from the controls) when 95% CI did not overlap with zero. Missing bars represent groups with a mortality of 100%.

This standardization of the parameter value of the treated sample (*P*_*T*_) with the mean value of the control treatment ($\bar{P_{C}}$) for each species allows species comparisons. Effects of flooding treatments on survived plants were considered significant (i.e. different from the controls) when 95% CI did not overlap with zero in Fig 2A–2C. As SLA values already represent a ratio, we skipped the RR procedure for this response variable.

Thereafter, one-way ANOVAs with the factor plant family were computed for every response variable, to account for potential phylogenetic effects (plant height: F = 0.692, p = 0.407; number of leaves: F = 21.14, p<0.001; biomass: F = 20.55, p<0.001; SLA: F = 0.012, p = 0.914). The residuals of these ANOVAs were used for the subsequent analyses. We calculated ANOVAs for each response variable (RR plant height, RR number of leaves, RR biomass and SLA) with the factors species (nested in microhabitat preference), seedling age and soil composition. To calculate the relative contribution of each factor or interaction to the total variance, we used the ratio: sum of squares of a factor/interaction divided by total sum of squares. Requirements to conduct ANOVA analyses (e.g. normality) were visually checked using diagnostic plots. All statistical analyses were carried out using R [59].

## Results

### Survival of plants

**Fig 3:**
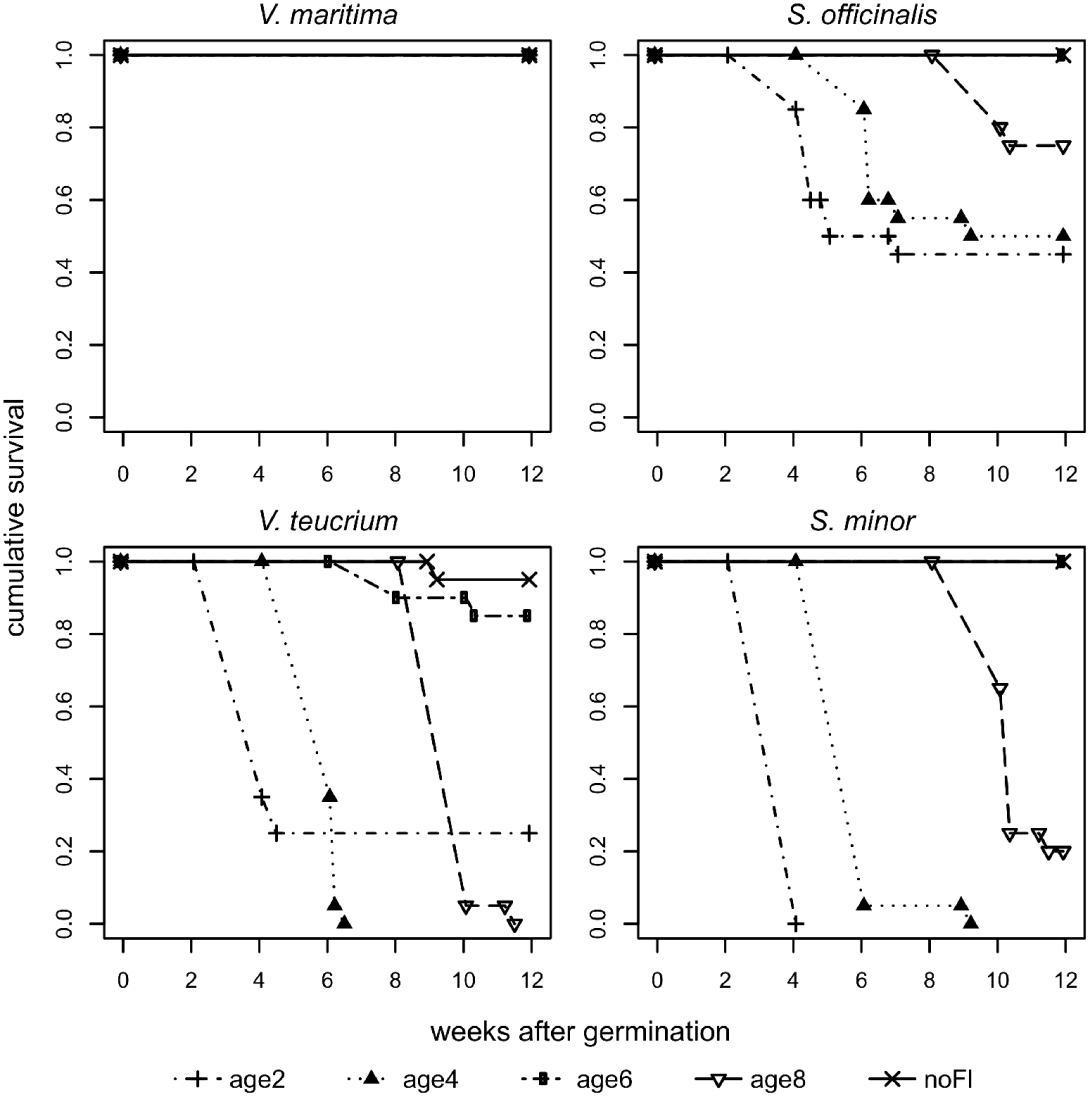
Effects of a 2-week flooding treatment on survival of four floodplain meadow plant species. Cumulative survival of *Veronica maritima* L., *Veronica teucrium* L., *Sanguisorba officinalis* L., and *Sanguisorba minor* Scop. after a 2-week flooding treatment, starting 2, 4, 6, and 8 weeks, respectively, after germination (age2-age8), and a control group with no flooding (noFl). age2, dot-dashed line & plus; age4, dotted line & filled triangle point up; age6, two-dashed line & circle; age8, long-dashed line & triangle point down; noFl, solid line & cross.

Of the 400 seedlings at the start of the experiment, 259 (64.75%) survived until the end. Survival across all treatments (4 seedling ages + control) was 14% in *V*. *teucrium*, 100% in *V*. *maritima*, 44% in *S*. *minor*, and 74% in *S*. *officinalis* (n = 100 plants per species). In the control group, i.e. no flooding treatment, overall only one individual of *V*. *teucrium* died (Fig 3).

Results of the survival analysis showed that the 2-week flooding treatment had a significant negative effect on cumulative survival of seedlings of three plant species (i.e. *S*. *minor*, *S*. *officinalis*, and *V*. *teucrium*) that belonged to the age groups age2, age4, and age8 (Table 1). All individuals of *V*. *maritima* survived until the end of the experiment; hence, our flooding treatments had no effect on this species (Fig 3). In the two species from dry microhabitats (*S*. *minor*, *V*. *teucrium*) two age groups showed 100% mortality (*S*. *minor*: age2, age4; *V*. *teucrium*: age4, age8). Contrarily, in species from wet microhabitats (*S*. *officinalis*, *V*. *maritima*) about half of the plants survived the flooding (e.g. *S*. *officinalis* lowest cumulative survival 0.45 and 0.5, Fig 3).

The risk of death in our experiment decreases with age, as indicated by the scale parameter of the accelerated failure time models of 0.31 being less than one. As expected, the significance of individual factors and interactions on survival showed a similar picture as the ANOVA analyses (Table 2). The effects of the flooding treatment showed similar impact on plant survival and on plant performance of survived plants. The survival of the species was affected by factors microhabitat (survival rates dry: 28.75%, wet: 83.75%) and seedling age (survival rates age2: 42.5%, age4: 37.5%, age6: 96.25%, age8: 48.75%), as well as species (nested in microhabitat) and species (nested in microhabitat) x seedling age interaction (Table 2).

**Table 2:** Performance of four floodplain meadow plant species after a 2-week flooding period.

	survival			plant height				number of leaves				biomass				SLA			
	df	dev	p	df	F	p	vc	df	F	p	vc	df	F	p	vc	df	F	p	vc
microhabitat (M)	1	96.1	**<0.001**	1	18.1	**<0.001**	5.6	1	53.2	**<0.001**	17.2	1	82.4	**<0.001**	20.9	1	13.5	**<0.001**	2.4
seedling age (A)	4	270.3	**<0.001**	3	23.1	**<0.001**	21.4	3	12	**<0.001**	11.7	3	22.2	**<0.001**	16.9	4	30.7	**<0.001**	22.1
soil (S)	1	1.5	0.214	1	0.6	0.427	0.2	1	0.9	0.347	0.3	1	0	0.976	0	1	2.2	0.135	0.4
species(microhabitat) [Sp(M)]	6	55.7	**<0.001**	2	1.2	0.302	0.7	2	0.6	0.532	0.4	2	4	**0.020**	2	2	45.4	**<0.001**	16.4
M x A	4	1.5	0.823	2	29.3	**<0.001**	18.1	2	22.5	**<0.001**	14.5	2	30.2	**<0.001**	15.3	2	16.9	**<0.001**	6.1
M x S	1	1.4	0.234	1	0.4	0.512	0.1	1	1.9	0.165	0.6	1	1.4	0.232	0.4	1	0.1	0.718	0
A x S	4	8.2	0.084	3	1.2	0.326	1.1	3	1.6	0.188	1.6	3	0.4	0.747	0.3	4	0.5	0.706	0.4
Sp(M) x A	24	56.4	**<0.001**	3	2.4	0.072	2.2	3	0.3	0.798	0.3	3	3	**0.030**	2.3	5	13	**<0.001**	11.7
Sp(M) x S	6	0.3	0.999	2	0.8	0.462	0.5	2	0.6	0.555	0.4	2	0.5	0.607	0.3	2	0.1	0.904	0
M x A x S	4	1.2	0.884	2	0.7	0.500	0.4	2	2.8	0.065	1.8	2	2.2	0.117	1.1	2	0.4	0.647	0.2
Sp(M) x A x S	24	1.0	1.000	3	1.4	0.239	1.3	3	0.9	0.443	0.9	3	1.2	0.314	0.9	5	0.6	0.696	0.5
Residuals	319			156			48.2	156			50.4	156			39.6	220			39.7

Effects of factors microhabitat, species nested in microhabitat, seedling age, and soil composition on the survival of all plant individuals, and on plant height (logarithmic response ratio), number of leaves (logarithmic response ratio), biomass (logarithmic response ratio), and specific leaf area (SLA) of survived plant individuals were tested performing a likelihood-ratio test of an accelerated failure time model using a Weibull error distribution and four ANOVA Analyses. df, degrees of freedom; dev, deviance; F, variance ratio; p, error probability; vc (%), relative contribution of individual factors and their interactions to total variance; p values < 0.05 are written in bold.

### Performance of plants

The performance of seedlings was not affected by differences in soil compositions (ANOVA analyses and accelerated failure time models: all p > 0.05). Negative flooding effects on plant growth i.e. reduced plant height, leaf number, and biomass production were significant for age groups age2 and age4 of all plants except *S*. *minor*, where both groups showed 100% mortality (Fig 2A–2C). This effect did not clearly decrease with age, but for the two species from wet microhabitats (i.e. *V*. *maritima* and *S*. *officinalis*) fitness of flooded plants was mostly not significantly different from the control for older seedlings (6 and 8 weeks after germination). Similarly, a slight but non-significant trend of increasing plant height with age was visible for *V*. *maritima* and *S*. *officinalis* (Fig 2A).

Microhabitat preference of the species, as reflected in Ellenberg indicator values (EIV) for moisture, had a significant impact on plants (over all four response variables, and on survival, Table 2): Plants from wet microhabitats showed less reduction in plant height and leaf number, higher biomass, and slightly higher SLA than plants from dryer microhabitats (all p < 0.001).

At the end of the experiment, the seedlings flooded at younger age (i.e. age groups age2 and age4) were smaller, had fewer leaves, and lower biomass than older seedlings (except for *S*. *minor*). Thus, also the factor seedling age explained a high amount of the total variance (vc, Table 2). Similarly, in the accelerated failure time models analysis, we found a significant effect of seedling age on the survival of the plants (Table 2).

Response of plants on flooding treatments was species-dependent, as indicated by the significance of species (nested in microhabitat) x seedling age interaction in accelerated failure time models and ANOVAs (Table 2). Inundated plants produced thinner leaves, which resulted in slightly higher SLA (not significant) compared to non-flooded plants from the control group (Fig 2D).

## Discussion

### The effects of age on the survival and performance of seedlings in response to flooding

Our experiment revealed that 2 weeks of flooding lowered survival of three of the four tested species (i.e. *S*. *officinalis*, *S*. *minor* and *V*. *teucrium*) and that survival increased with the age of the seedlings, as risk of death decreased. Our first hypothesis that under a 2-week flooding period, older seedlings perform better than younger seedlings, therefore was accepted. These results are in line with a study by Nabben et al. [27], who found that juvenile plants of three *Rumex* species showed lower survival (approx. by factor four) than mature plants. In accordance to this study, we expected survival increasing with age of the seedlings over individual age groups. However, for the oldest group, with flooding start at an age of 8 weeks after germination, survival was lower than expected. This outcome can be explained by particularly high temperatures during this flooding treatment (age8, Fig 1B). Summer floods may result in heating of the slow flowing, ponded water on the floodplain meadows and this probably also happened to our experimental plants. This rise in water temperature most likely forced additional damage of flooded plants, as warm temperatures increase enzyme activity and limit oxygen solubility [60]. Detrimental flooding effects on mature grasses are known to be greater at high water temperature (30°C) compared to low temperature (10°C) floods [61]. Hence, summer floods are likely more harmful than flooding events earlier in the year. Likewise, Van Eck et al. [23] showed that mainly summer flooding defines zonation of plants on flood meadows. Our data may suggest an age threshold for flood meadow species from wet microhabitats between 4 and 6 weeks after which the negative effects of a 2-week flooding event appears to be significantly reduced. Likewise, Hidding et al. [44] recently suggested that flooding outcome (i.e. promotion of plant growth vs. severe damaging of plants) depends strongly on the timing of flooding. In their experiment, wetland plants, with an age of approx. 5 weeks at the start of the flooding treatment, showed elongation of plant growth (7 out of 8 species) but also unclear responses in horizontal expansion and biomass production after flooding. Also for *Phragmites australis* seedlings the tolerance to submergence increased with age [25], hence this effect may be ubiquitous for plants from riparian ecosystems.

### The effects of substrate on the survival and performance of seedlings in response to flooding

Differences in soil composition (i.e. soil:sand ratio of 3:1 vs. 1:1) had no effect on the response variables (Table 2). Thus, our second hypothesis that under a 2-week flooding period, the performance of the seedlings decreases with increased sand content, was rejected. Interestingly, Lenson et al. [62] showed that wetland species produce more biomass on soils with organic sediments compared to mineral sediments. They concluded that this was caused by the low nutrient availability in the mineral-sediment soil. In our study, maintaining nutrient equivalence in the two soil:sand ratio groups resulted in similar plant performance, which supports the conclusions of Lenson et al. [62]. Likewise, in a study on floodplains along the Middle Elbe, sand content only weakly explains species composition [63].

### Differences in the survival and performance of seedlings from wet vs. dry microhabitats in response to flooding

We found evidence that under flooding treatment, species preferring wet microhabitats grow higher and survive longer compared to species from dry microhabitats. This confirmed our third hypothesis that under a 2-week flooding period, plant species from wet microhabitats perform better compared to those of dry microhabitats. Higher survival and plant growth of *V*. *maritima* compared to *S*. *officinalis* within the wet microhabitat is consistent with differences in Ellenberg indicator values between the two species (Table 1) [47]. More generally, our findings cohere with the expectations that flood sensitive species are located on higher parts of the floodplain where flooding impacts are limited. In contrast, flood tolerant species survive at areas with more frequent flooding at lower elevations [13,23,64]. Likewise, leaf thickness of plants varies between species with different microhabitat preferences. SLA of plants adapted to wet microhabitats is higher than of plants from dry microhabitats (Table 2). Also Koike et al. [65] found contrasting SLA values for birch species with different microhabitat preferences under wet soil moisture conditions. In addition, our result that leaf plasticity differs between treatment and control (i.e. SLA of flooded plants is slightly higher than for plants from control group, Fig 2D) is in accordance with previous findings. Plants under submergence develop thinner, elongated leaves and therefore show increased SLA (for review see [66]).

### Synopsis for restoration management

From a restoration ecological perspective, our finding that seedlings of flood-meadow species respond differently to flooding events at young age show the difficulties of measures that aim to reestablish floodplain vegetation (e.g. via the transfer of seed-containing plant material) [67]. The forecasted increase in extreme discharge events owing to climate change will simultaneously raise the risk for restoration measures in terms of costs and logistic effort. To increase restoration success, habitat requirements of the individual target plant species and microhabitat characteristics of restoration sites have to match. Habitat-suitability maps on a microhabitat scale for the target species could incorporate all these factors and enhance restoration planning [68]. In case of planning large-scale restoration projects, especially regarding ecological (i.e. prescribed) flooding, our findings should also be taken into account. After a floodplain restorations, the schedule of gate openings at ecological flooding sites should be adapted to germination timing of target species to enhance survival and establishment of target species.

### Conclusions

In conclusion, our results demonstrated the importance of seedling age and microhabitat preference of plants on their flooding tolerance, whereas soil composition had no effect. Based on our data, we predict that for future restoration measures of floodplain meadows (e.g. the transfer of freshly cut seed-containing plant material) the restoration success after a medium flooding event will be higher, if the plants have reached the critical threshold age of about 6 weeks after germination. Besides, flooding in summer may also lead to stronger damages of plants due to higher floodwater temperatures. Vegetation of floodplain meadows indeed is affected by seasonal flow patterns (for review see [69]). All these aspects demonstrate the increasing vulnerabilities of floodplain meadow species under the predicted alterations of climatic and thus hydrologic conditions [39]. Hence, the complexity regarding timing of floodplain meadow restorations and of conservation planning in floodplain landscapes in general is increasing.

## Supporting information

S1 Fig Photograph of experimental setup. Photograph showing experimental plant pots placed inside of 1.2L transparent polypropylene cups and distributed randomly on a paved area at the research station Linden-Leihgestern (Hesse, Germany, UTM: 32U 478260 5598300) in May 2015. Photo: Johannes P. Gattringer.
(PNG)

S1 Dataset Dataset of the experiment. (XLSX)
