# Supplementary figures and images for: Flooding tolerance of four floodplain meadow species depends on age
Source: PLoS One. 2017 May 3;12(5):e0176869. doi: 10.1371/journal.pone.0176869 (PMC5415089; doi:10.1371/journal.pone.0176869)

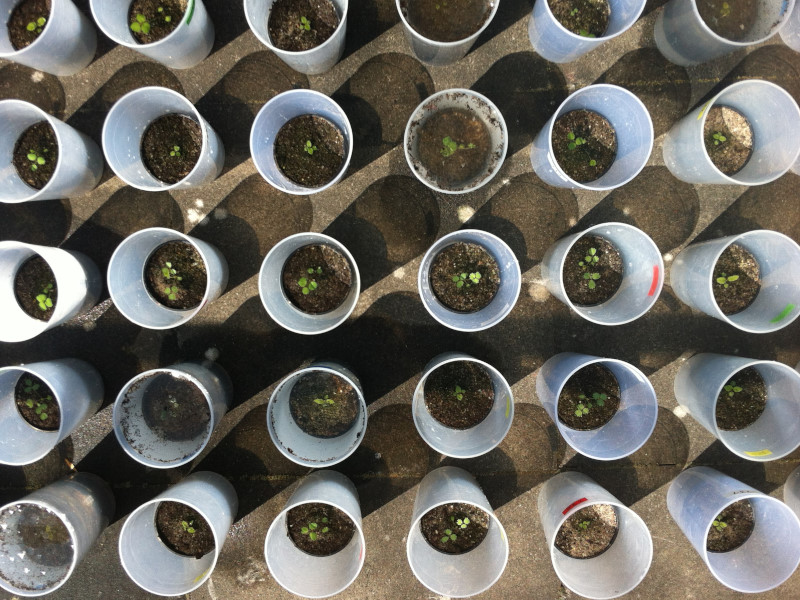

Supplement: S1 Fig — Photograph showing experimental plant pots placed inside of 1.2L transparent polypropylene cups and distributed randomly on a paved area at the research station Linden-Leihgestern (Hesse, Germany, UTM: 32U 478260 5598300) in May 2015. Photo: Johannes P. Gattringer. (PNG) [file pone.0176869.s001.png]
